# Association of fresh vegetable and salt-preserved vegetable consumptions with estimated glomerular filtration rate

**DOI:** 10.1186/s12882-023-03353-5

**Published:** 2023-12-12

**Authors:** Haiqing Zheng, Huixian Li, Liyan Pan, Lianting Hu, Xuanhui Chen, Jiaxin Hou, Huiying Liang

**Affiliations:** Medical Big Data Center, Guangdong Provincial People’s Hospital, Guangdong Academy of Medical Sciences, Southern Medical University, No.106, Zhongshan Er Road, Guangzhou, Guangdong China

**Keywords:** Association, Fresh vegetables, Salt-preserved vegetables, Estimated glomerular filtration rate, Oldest subjects

## Abstract

**Objective:**

This study aimed to investigate the relationship between the consumption of fresh and salt-preserved vegetables and the estimated glomerular filtration rate (eGFR), which requires further research.

**Methods:**

For this purpose, the data of those subjects who participated in the 2011–2012 and 2014 surveys of the Chinese Longitudinal Healthy Longevity Survey (CLHLS) and had biomarker data were selected. Fresh and salt-preserved vegetable consumptions were assessed at each wave. eGFR was assessed using the Chronic Kidney Disease Epidemiology Collaboration (CKD-EPI) equation based on plasma creatinine. Furthermore, a linear mixed model was used to evaluate associations between fresh/salt-preserved vegetables and eGFR.

**Results:**

The results indicated that the median baseline and follow-up eGFRs were 72.47 mL/min/1.73 m² and 70.26 mL/min/1.73 m², respectively. After applying adjusted linear mixed model analysis to the data, the results revealed that compared to almost daily intake, occasional consumption of fresh vegetables was associated with a lower eGFR (β=-2.23, 95% CI: -4.23, -0.23). Moreover, rare or no consumption of salt-preserved vegetables was associated with a higher eGFR (β = 1.87, 95% CI: 0.12, 3.63) compared to individuals who consumed salt-preserved vegetables daily.

**Conclusion:**

Fresh vegetable consumption was direct, whereas intake of salt-preserved vegetables was inversely associated with eGFR among the oldest subjects, supporting the potential benefits of diet-rich fresh vegetables for improving eGFR.

## Introduction

The prevalence of chronic kidney disease (CKD) generally increases with age owing to nephron loss and a decline in the glomerular filtration rate (GFR). The estimated GFR (eGFR) assumes a widely recognized role as a marker that plays a pivotal role in evaluating overall kidney health and diagnosing various renal disorders. Its significance is rooted in its capacity to offer clinicians a quantitative measurement of the glomeruli’s filtration efficiency, thereby assisting in the early detection and continuous monitoring of conditions like CKD, acute kidney injury, and other renal pathologies. The elderly population with CKD exhibits an elevated risk of cardiovascular disease [1] and end-stage renal disease [2], which are major causes of mortality [3]. Delaying CKD progression has a significant beneficial impact on patients and the healthcare system. Diet plays a pivotal role in promoting health, however, some dietary habits can exacerbate the acceleration of kidney function decline and the incidence of CKD [4]. Dietary patterns characterized by high consumption of plant-based foods, vegetables, fruits, and fresh foods are thought to be inversely associated with CKD [5–7]. However, it is unknown whether a single component of these diets, such as a higher intake of vegetables, is associated with improved kidney function. Vegetarian diets may decrease the production of uremic toxins and reduce salt intake and acid overload. An early study conducted in older Americans with CKD stage 4 suggested that increasing fruit and vegetable consumption might lower net endogenous acid excretion [8], which minimizes individual nephron workload and slows the progressive loss of kidney function [9, 10]. However, a prospective cohort study in a large Dutch population found no association between vegetable intake and renal function decline [11]. Such inconsistencies in previous studies warrant a re-examination of the associations between vegetable intake and kidney function in the Asian elderly population.

In contrast, high salt intake may increase urine protein levels and is recognized as a risk factor for reduced kidney function [12–14], mainly by adversely affecting blood pressure and vascular health [15]. The primary food sources of sodium are seasonings and vegetables such as salt-preserved vegetables. Salting is one of the most common and oldest methods of food preservation in China and is appreciated by people, especially the elderly. A study of Chinese adults suggested that excessive salt-preserved food, but not other unhealthy dietary behaviors, deteriorate renal function in patients with diabetes [16]. However, in a study of 9229 Korean participants, fermented vegetables were not significantly associated with CKD risk [5]. In addition, a study performed with the US population from 2001 to 2006 revealed that higher sodium intake was associated with lower odds of CKD [17]. Hence, there is inconclusive evidence regarding whether dietary sodium consumption can affect and how sodium affects kidney function decline.

Given the limited and inconsistent evidence on the relationship between fresh and salt-preserved vegetables and renal function, particularly among the elderly, we examined the prospective association between the two vegetable types and renal function in older people in China.

## Methods

### Design and participants

The data used in this longitudinal study were derived from the Chinese Longitudinal Healthy Longevity Survey (CLHLS), the details of which have been previously described [18]. Since 1998, seven rounds of follow-up surveys were conducted in 2000, 2002, 2005, 2008–2009, 2011–2012, 2014, and 2018–2019. The response rate for each survey wave was approximately 90%. The CLHLS replaced deceased participants with new participants in the follow-up waves to maintain a sufficient sample size. In addition, blood tests were conducted, and blood samples were collected from consenting participants during three waves: 2008–2009, 2011–2012, and 2014.

Due to the notable loss of participants during the period spanning from 2008 to 2014, resulting in an escalated attrition rate, introduces the possibility of significant bias exerting influence upon the study’s findings. The current study utilized data from 2011 to 2014 wave of the CLHLS. The study’s inclusion criteria encompassed participants aged 60 to 113 who had blood test data available for the estimation of kidney function. Participants without information about their dietary habits at baseline or follow-up blood tests for kidney function, as well as individuals with baseline renal disease (self-reported diagnosis by a doctor at a level II or above hospital), were excluded from the study.

### Fresh vegetable and salt-preserved vegetable intake

Information on usual dietary habits was collected through face-to-face interviews with trained research staff. The question about fresh vegetable intake consisted of “how often do you eat fresh vegetables at present?” The answer choices included “almost daily”, “almost daily except in winter”, “occasionally”, and “rarely or never”. We considered consuming fresh vegetables “almost daily” and “almost daily except in winter” as “high fresh vegetable intake”, “occasionally” and “rarely or never” as “low fresh vegetable intake”. The question about salt-preserved vegetables included “how often do you eat salt-preserved vegetables at present?” the answer choices included “almost daily”, “at least once weekly”, “at least once per month”, “occasionally” and “rarely or never”. We combined the responses of “at least once weekly”, “at least once per month” and “occasionally” and labeled them as “occasionally”. We considered consuming salt-preserved vegetables “almost daily” as “high salt-preserved vegetable intake”, “occasionally” and “rarely or never” as “low salt-preserved vegetable intake”.

### Estimated glomerular filtration rate

Venous blood was collected in heparin anticoagulant vacuum tubes and centrifuged at 20 °C at 2500 rpm for 10 min. Plasma was isolated and frozen at -20 °C, shipped on wet ice to the central laboratory at Capital Medical University in Beijing and stored at -80 °C until further analysis. The serum creatinine levels were measured using the picric acid method. All the laboratories engaged in the investigation underwent a standardization and certification program. Both the baseline and follow-up measurements were conducted within the same laboratory setting [19].

The eGFR was calculated using the Chronic.

Kidney Disease-Epidemiology Collaboration equation [20]:

eGFR = 141 × min(Scr/κ, 1)^α^ × max(Scr/κ, 1)^−1.209^ × 0.993^Age^ × 1.018 [if female] × 1.159 [if black].

Where Scr = serum creatinine, κ is 0.7 for females and 0.9 for males, α is -0.329 for females and − 0.411 for males, min indicates the minimum of Scr/κ or 1, and max indicates the maximum of Scr/κ or 1.

### Covariates

Covariates in this study comprised sociodemographic information: age (year), sex (men and women), education level (no schooling/some schooling, defined as one year of schooling or more), body mass index (kg/m^2^), residence (urban or rural), marital status (married or unmarried), economic status (rich, fair, poor); lifestyle-related variables: smoking status (current smoker, former smoker, or nonsmoker), alcohol consumption (current drinker, former drinker, or nondrinker), exercise status (current, former, or non-exerciser), vitamins consumption (vitamins A/C/E); health status involving a self-reported history of hypertension (yes or no), diabetes mellitus (yes or no), pneumonia (yes or no) and heart disease (yes or no), and other parameters such as albumin, urea nitrogen, urea acid, lipid profiles (triglycerides, high-density lipoprotein cholesterol (HDL-C), low-density lipoprotein cholesterol (LDL-C) and total cholesterol, and high-sensitivity C-reactive protein.

### Statistical analysis

We first described the baseline characteristics of the study population by vegetable and salt-preserved vegetable intake. Continuous variables were tested for normality and for the homogeneity of variance using the Kolmogorov-Smirnov test and Levene homogeneity of variance test, respectively. Analysis of variance (ANOVA) was used for normally distributed variables with equal population variance, and the non-parametric (Kruskal-Wallis) test was used for non-parametric variables. Categorical variables are demonstrated as frequencies with proportions and were compared using Pearson’s Chi-square test. We used linear mixed model to investigate eGFR and their relationships with fresh and salt-preserved vegetables. To further expand our findings, we explored potential effect modification by fresh and salt-preserved vegetable intake and performed a subgroup analysis. Three models were run for each primary independent variable: (1) a crude model (model 1); (2) a model adjusted for age, sex, residence, married status, level of education, economics, physical activity, BMI, fruit intake, meat intake, fish intake, egg intake, vitamin intake, smoking, drinking, hypertension, diabetes, heart disease, respiratory disease, and urinary albumin (model 2); and (3) fresh vegetable intake (for salt-preserved vegetable analysis) or salt-preserved vegetable intake (for fresh vegetable analysis) to investigate the possible mediating roles of each other (model 3). To explore the impact of vegetables on eGFR, we employed Spearman correlation analysis to examine the correlations between vegetable consumption, hs-CRP, and eGFR. Overall, missing data were uncommon, no more than 5% of the data for any variable, and multiple imputations were used to reduce bias due to missing covariate data. All statistical analyses were performed using R (version 3.6.1), with p-values less than 0.05 considered statistically significant.

## Results

### Characteristics of the study population

**Fig. 1:**
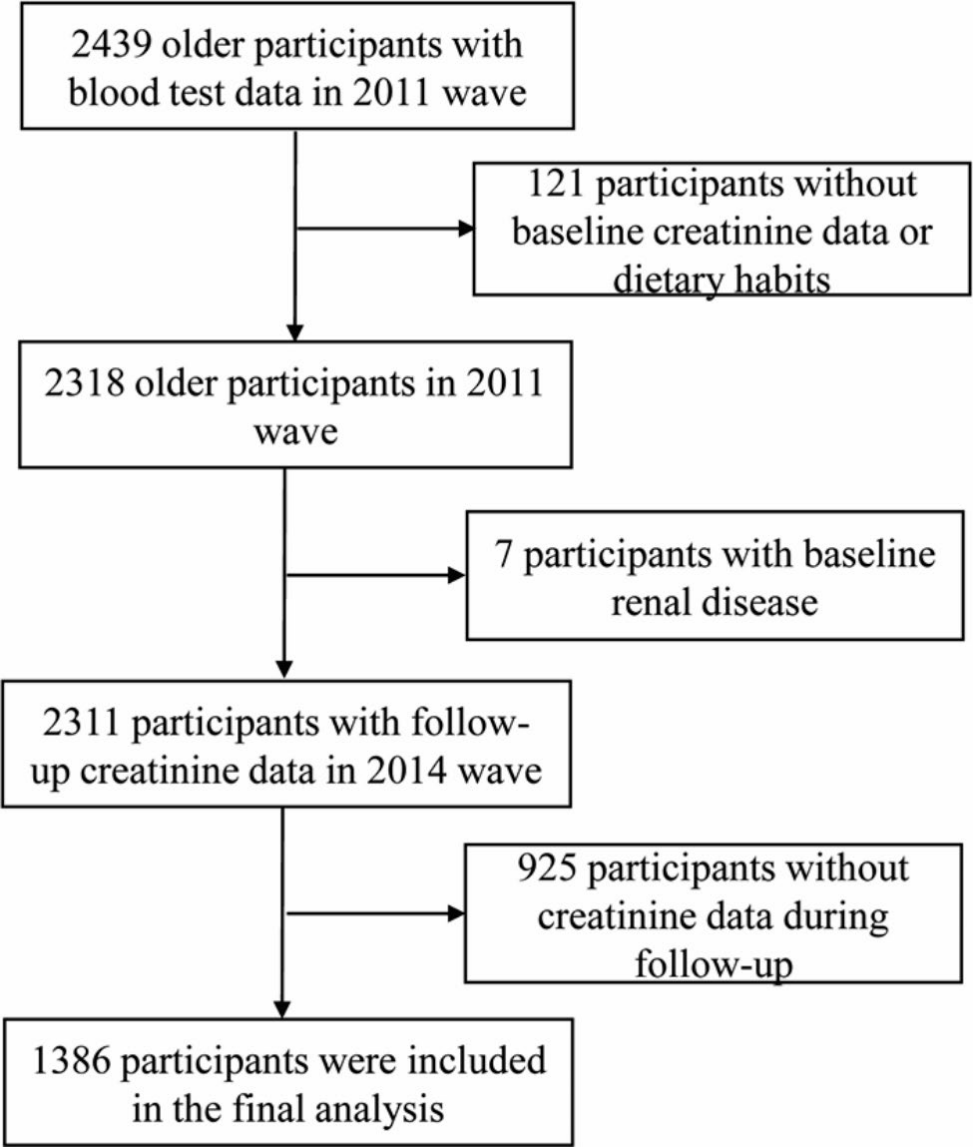
Flowchart of study population

A flowchart outlining participant enrollment in this study is provided in Fig. 1, and the baseline characteristics of participants according to the frequency of fresh vegetable intake and the frequency of salt-preserved vegetable intake are reported in Table 1. At baseline, the median (IQR) age of the selected population was 82.00 (72.00–91.00) years, among which 51.4% were females. The baseline eGFR was calculated to be 72.47 mL/min/1.73 m², whereas the follow-up eGFR was noted as 70.26 mL/min/1.73 m^2^. Compared with participants who consumed fresh vegetables almost daily, those who consumed fresh vegetables almost daily except winter, occasionally and rarely/never were significantly more likely to be older, illiterate, living in rural, general economic status, unmarried, with no exercise, having lower albumin, higher triglycerides, high-density lipoprotein cholesterol, low-density lipoprotein cholesterol, and lower diastolic pressure. The characteristics in the salt-preserved vegetable group were similar to those in the fresh group.

**Table 1:** Baseline characteristics of 1386 participants according to fresh and salt-preserved vegetable intakes

	Fresh vegetable intakes						Salt-preserved vegetable intakes			
Variables	Almost everyday (n = 674)	Except winter(n = 549)	Occasionally (n = 107)	Rarely or never(n = 56)	*P*		Almost everyday (n = 186)	Occasionally (n = 783)	Rarely or never(n = 417)	*P*
Age, years	81.0(71.0–90.0)	80.0(71.0–91.0)	89.0(80.0–96.0)	88.0(80.3–97.8)	< 0.001		79.0(71.0–89.0)	81.0(71.0–91.0)	84.0(74.0–94.5)	< 0.001
Sex					0.008					0.033
Male	323(47.9)	288(52.5)	44(41.1)	18(32.1)			88(47.3)	403(51.5)	182(43.6)	
Female	351(52.1)	261(47.5)	63(58.9)	38(67.9)			98(52.7)	380(48.5)	235(56.4)	
BMI,kg/m^2^	21.73 ± 4.35	21.49 ± 3.57	20.91 ± 3.18	21.34 ± 4.38	0.226		22.31 ± 3.96	21.67 ± 3.92	20.99 ± 4.02	< 0.001
Education					<0.001					<0.001
No schooling	345(51.2)	334(60.8)	81(75.7)	40(71.4)			89(47.8)	435(55.6)	276(66.2)	
Some schooling	329(48.8)	215(39.2)	26(22.3)	16(28.6)			97(52.2)	348(44.4)	141(33.8)	
Residence					0.008					0.135
Urban	132(19.6)	78(14.2)	9(8.4)	9(16.1)			23(12.4)	141(18.0)	64(15.3)	
Rural	542(80.4)	471(85.8)	98(91.6)	47(83.9)			163(87.6)	642(82.0)	353(84.7)	
Economic status					<0.001					0.013
Rich	126(18.7)	67(12.2)	6(5.6)	3(5.4)			41(22.0)	112(14.3)	49(11.8)	
Fair	495(73.4)	432(78.7)	80(74.8)	49(87.5)			125(67.2)	603(77.0)	328(78.7)	
Poor	53(7.9)	50(9.1)	21(19.6)	4(7.1)			20(10.8)	68(8.7)	40(9.6)	
Married status					<0.001					0.050
Married	343(50.9)	265(48.3)	32(29.9)	12(21.4)			102(54.8)	366(46.7)	184(44.1)	
Unmarried	331(49.1)	284(51.7)	75(70.1)	44(78.6)			84(45.29)	417(53.3)	233(55.9)	
Fruit intake					< 0.001					< 0.001
Almost everyday	287(42.6)	229(41.7)	6(5.6)	17(17.9)			72(38.7)	329(42.0)	131(31.4)	
Occasionally	223(33.1)	231(42.1)	78(72.9)	7(12.5)			64(34.4)	321(41.0)	154(36.9)	
Rarely or never	164(24.3)	89(16.2)	23(21.5)	39(69.6)			50(26.9)	133(17.0)	132(31.7)	
Meat intake					< 0.001					0.001
Almost everyday	277(41.1)	104(18.9)	21(19.6)	23(41.1)			53(28.5)	227(29.0)	145(34.8)	
Occasionally	375(55.6)	418(7.1)	78(72.9)	32(57.1)			120(64.5)	536(68.5)	247(59.2)	
Rarely or never	22(3.3)	27(4.9)	8(7.5)	1(1.8)			13(7.0)	20(2.6)	25(6.0)	
Fish intake					< 0.001					< 0.001
Almost everyday	79(11.7)	19(3.5)	3(2.8)	3(5.4)			21(11.3)	48(6.1)	35(8.4)	
Occasionally	533(79.1)	467(85.1)	92(86.0)	42(75.0)			149(80.1)	670(85.6)	315(75.5)	
Rarely or never	62(9.2)	63(11.5)	12(11.2)	11(19.6)			16(8.6)	65(8.3)	67(16.1)	
Egg intake					< 0.001					< 0.001
Almost everyday	234(34.7)	198(36.1)	21(19.6)	19(33.9)			78(41.9)	258(33.0)	136(32.6)	
Occasionally	377(55.9)	330(60.1)	75(70.1)	29(51.8)			98(52.7)	487(62.2)	226(54.2)	
Rarely or never	63(9.3)	21(3.8)	11(10.3)	8(14.3)			10(5.4)	38(4.9)	55(13.2)	
Vitamin intake					0.771					< 0.001
Almost everyday	17(2.5)	15(2.7)	3(2.8)	1(1.8)			5(2.7)	16(2.0)	15(3.6)	
Occasionally	72(10.7)	53(9.7)	11(10.3)	2(3.6)			8(4.3)	103(13.2)	27(6.5)	
Rarely or never	585(86.8)	481(87.6)	93(86.9)	53(94.6)			173(93.0)	664(84.8)	375(89.9)	
Smoking					0.097					0.308
Current	148(22.0)	107(19.5)	16(15.0)	4(7.1)			43(23.0)	162(20.7)	70(16.8)	
Past	58(8.6)	53(9.7)	7(6.5)	6(10.7)			18(9.6)	71(9.1)	35(8.4)	
Never	468(69.4)	389(70.9)	84(78.5)	46(82.1)			126(67.4)	550(70.2)	312(74.8)	
Drinking status					0.069					<0.001
Current	126(18.7)	103(18.8)	9(8.4)	5(8.9)			44(23.7)	148(18.9)	51(12.2)	
Past	44(6.5)	36(6.6)	5(4.7)	3(5.4)			19(10.2)	48(6.1)	21(5.0)	
Never	504(74.8)	410(74.7)	93(86.9)	48(85.7)			123(66.1)	587(75.0)	345(82.7)	
Exercise					0.001					0.477
Current	141(20.9)	70(12.8)	14(13.1)	3(5.4)			32(17.12)	134(17.1)	62(14.9)	
Past	13(1.9)	13(2.4)	1(0.9)	1(1.8)			2(1.1)	14(1.8)	12(2.9)	
Never	520(77.2)	466(84.9)	93(86.0)	52(92.9)			152(81.7)	635(81.1)	343(82.3)	
Hypertension					0.713					0.342
Yes	405(60.1)	325(59.2)	61(57.0)	37(66.1)			111(59.7)	456(58.2)	261(62.6)	
No	269(39.9)	224(40.8)	46(43.0)	19(33.9)			75(40.3)	327(41.8)	158(37.4)	
Diabetes mellitus					0.493					0.536
Yes	53(7.9)	36(6.6)	11(10.3)	3(5.4)			15(8.1)	62(7.9)	26(6.2)	
No	621(92.1)	513(93.4)	96(89.7)	53(94.6)			171(91.9)	721(92.1)	391(93.8)	
Pneumonia					0.657					0.463
Yes	50(7.4)	45(8.2)	8(7.5)	2(3.6)			12(6.5)	56(7.2)	37(8.9)	
No	624(92.6)	504(91.8)	99(92.5)	54(96.4)			174(93.5)	727(92.8)	380(91.1)	
Heart disease					0.095					0.001
Yes	58(8.6)	39(7.1)	13(12.1)	1(1.8)			27(14.5)	51(6.5)	33(7.9)	
No	616(91.4)	510(92.9)	94(87.9)	55(98.2)			159(85.5)	732(93.5)	384(92.1)	
Albumin, g/L	41.3(37.9–44.4)	40.9(38.2–43.8)	39.7(36.9–41.8)	39.7(3.7–42.7)	< 0.001		42.8(39.6–46.0)	40.9(37.8–44.0	40.4(37.4–43.0)	< 0.001
Urinary albumin	4.4(0.7–16.7)	4.9(0.9–13.6)	5.3(1.2–11.4)	4.9(1.0–9.4)	0.914		4.9(0.9–19.6)	4.7(0.8–15.2)	4.7(0.7–11.2)	0.586
Hs-CRP, mg/L	0.8(0.4–2.1)	0.9(0.4–2.1)	1.2(0.5–3.0)	0.9(0.5–1.2)	0.046		0.7(0.4–1.5)	0.9(0.4–2.3)	0.9(0.4–2.5)	0.008
Urea, mmol/L	6.4(5.2–7.6)	6.5(5.4–7.8)	6.6(5.4–7.6)	6.4(5.7–7.3)	0.535		6.1(5.0–7.5)	6.5(5.5–7.7)	6.4(5.2–7.6)	0.066
Urea acid, umol/L	286.8(239.4–349.2)	271.9(220.1–331.4)	273.7(227.3–323.9)	260.8(221.9–348.2)	0.005		273.6(218.4–333.7)	282.9(234.6–344.0)	273.7(230.3–338.9)	0.195
Triglycerides, mmol/L	0.9(0.6–1.3)	0.8(0.6–1.2)	0.8(0.6–1.1)	0.8(0.5–1.1)	0.004		0.8(0.6–1.2)	0.9(0.6–1.2)	0.80(0.6–2.5)	0.483
HDL-C, mmol/L	1.3(1.1–1.6)	1.2(1.0–1.5)	1.2(1.0–1.4)	1.2(1.0–1.5)	0.031		1.3(1.1–1.6)	1.2(1.0–1.5)	1.3(1.1–1.5)	0.047
LDL-C, mmol/L	2.5(1.9–3.0)	2.6(2.1–3.1)	2.5(2.2–2.9)	2.6(2.2–3.3)	0.047		2.5(2.1–3.0)	2.5(2.0–3.1)	2.5(2.0–3.1)	0.350
Total cholesterol, mmol/L	4.3(3.7–5.0)	4.3(3.7–5.0)	4.2(3.7–4.8)	4.4(3.6–5.0)	0.338		4.4(3.7–4.9)	4.3(3.7–4.9)	4.3(3.7–5.0)	0.388
SBP, mmHg	140.0(125.0–156.0)	140.0(122.5–152.1)	137.5(122.5–149.0)	143.0(127.5–160.0)	0.179		140.0(125.3–159.0)	139.0(125.0–154.8)	140.0(125.0–152.5)	0.386
DBP, mmHg	80.0(70.0–90.0)	80.0(70.0–90.0)	80.0(72.5–90.0)	80.0(75.0–90.0)	0.011		80.0(75.0–90.0)	80.0(73.0–90.0)	80.0(70.0–90.0)	0.759
Glucose, mmol/L	4.3(3.5–5.1)	4.5(3.8–5.1)	4.5(3.7–5.2)	4.3(3.6–4.9)	0.008		4.4(3.7–4.9)	4.5(3.8–5.2)	4.3(3.5–5.1)	0.005
Baseline_eGFR, ml/min /1·73 m^2^	73.1(59.3–83.7)	73.5(60.8–85.7)	64.0(54.3–75.0)	66.1(51.3–81.1)	< 0.001		77.6(62.2–87.1)	72.6(57.1–83.8)	70.3(56.4–80.9)	< 0.001
Follow-up_eGFR, ml/min/1·73 m^2^	71.2(58.8–83.6)	71.1(57.8–83.7)	67.8(54.9–77.4)	57.9(47.8–73.3)	< 0.001		71.6(59.5–82.7)	69.6(56.8–83.2)	70.8(58.0–82.0)	0.549

BMI, body mass index; CRP, C-reactive protein; HDL-C, high-density lipoprotein cholesterol; LDL-C, low-density lipoprotein cholesterol; SBP, systolic blood pressure; DBP, diastolic blood pressure; eGFR, estimated glomerular filtration rate

### Associations of fresh and salt-preserved vegetable consumptions with eGFR

Table 2 depict the associations between the intake of fresh and salt-preserved vegetables and eGFR. In the multivariable-adjusted model, compared to participants who consumed fresh vegetables almost daily, those who consumed occasionally had a lower eGFR (β=-2.23, 95% CI: -4.23, -0.23). However, owing to the small sample size, no association was found between rarely/never consumed fresh vegetables and eGFR. On the contrary, participants who rarely/never consumed salt-preserved vegetables were associated with a higher eGFR (β = 1.87, 95% CI: 0.12, 3.63), compared with those who consumed salt-preserved vegetables almost daily. Furthermore, correlation analysis revealed correlation coefficients of 0.022 (P = 0.048) between vegetables and hs-CRP, and 0.151 (P < 0.001) between hs-CRP and eGFR.

**Table 2:** Regression coefficients (95%CIs) for the associations between fresh and salt preserved vegetable intakes and eGFR

Variables	Model 1	Model 2	Model 3
Fresh Vegetables			
Almost everyday	Reference	Reference	Reference
Except winter	0.18(-1.20, 1.56)	-1.18(-2.36, 0.01)	-1.16(-2.33, 0.02)
Occasionally	-5.31(-7.64, -2.98)	-2.17(-4.17, -0.17)	-2.23(-4.23, -0.23)
Rarely or never	-6.97(-10.45, -3.50)	-2.89(-5.84, 0.07)	-3.26(-6.22, -0.32)
Salt-preserved vegetables			
Almost everyday	Reference	Reference	Reference
Occasionally	-2.29(-4.18, -0.40)	-1.03(-2.61, 0.54)	-0.82(-2.40, 0.75)
Rarely or never	-1.64(-3.72, 0.43)	1.58(-0.16, 3.33)	1.87(0.12, 3.63)

Model 1, no adjustment; model 2. Age, sex, residence, married status, level of education, economics,

physical activity, BMI, fruit intake, meat intake, fish intake, urinary albumin, egg intake, vitamin intake,

smoking, drinking, hypertension, diabetes, heart disease and respiratory disease; Model 3 vegetable

(for salt preserved vegetables analysis) or salt preserved vegetables (for vegetables analysis)

### Associations of a combination of fresh and salt-preserved vegetables with eGFR

All participants were categorized into four sub-groups based on fresh vegetables and salt-preserved vegetables, that is, low fresh vegetable intake + high salt-preserved vegetable intake (n = 16), low fresh vegetable intake + low salt-preserved vegetable intake (n = 205), high fresh vegetable intake + high salt-preserved vegetable intake (n = 174), and high fresh vegetable intake + low salt-preserved vegetable intake (n = 991). As illustrated in Table 3, compared to the group with low fresh and high salt-preserved vegetable intake, the other three groups displayed higher eGFR. In participants with high salt-preserved vegetable intake, a low eGFR (β=-5.71, 95% CI: -11.34, -0.73) was found in participants who had a lower intake of fresh vegetables. However, no statistically significant associations were found in the low salt-preserved vegetable intake, low fresh vegetable intake, and high fresh vegetable intake subgroups.

**Table 3:** Associations between fresh vegetable intake, salt preserved vegetable intake, and eGFR under different stratifications

		n	Model 1			Model 2		
Fresh vegetables + salt preserved vegetables		In this "n" column, the original numbers represented cases of participants(each participant having two follow-ups), and now they have been changed to frequencies. The modified data represents the final results						
Low fresh vegetable intake + high salt-preserved vegetable intake		16	Reference			Reference		
Low fresh vegetable intake + low salt-preserved vegetable intake		205	13.77(6.91, 20.62)			6.02(0.37, 11.67)		
High fresh vegetable intake + high salt-preserved vegetable intake		174	6.46(-0.42, 13.35)			4.16(-1.51, 9.83)		
High fresh vegetable intake + low salt-preserved vegetable intake		991	11.51(4.84, 18.18)			5.62(0.11, 11.13)		
Fresh vegetables								
*Low fresh vegetable intake*								
High salt-preserved vegetable intake		16	Reference			Reference		
Low salt-preserved vegetable intake		205	6.51(0.33, 12.69)			3.65(-1.96, 9.26)		
*High fresh vegetable intake*								
High salt-preserved vegetable intake		174	Reference			Reference		
Low salt-preserved vegetable intake		991	-2.26(-4.17, -0.34)			-0.32(-1.92, 1.27)		
Salt-preserved vegetables								
*Low salt-preserved vegetable intake*								
High fresh vegetable intake		991	Reference (The next three columns shifted one grid to the right)			Reference		
Low fresh vegetable intake		205	-5.04(-7.08, -3.01)			-1.50(-3.25, 0.25)		
*High salt-preserved vegetable intake*								
High fresh vegetable intake		174	Reference			Reference		
Low fresh vegetable intake		16	-13.7(-20.22, -7.26)			-5.71(-11.34, -0.73)		

Model 1, no adjustment; model 2. Age, sex, residence, married status, level of education, economics, physical activity,

BMI, fruit intake, meat intake, fish intake, urinary albumin, egg intake, vitamin intake, smoking, drinking, hypertension,

diabetes, heart disease and respiratory disease

## Discussion

In this population-based cohort of the elderly, it was observed that lower consumption of fresh vegetables was associated with a lower eGFR. Nevertheless, this association was significant only in those with high salt-preserved vegetable intake. Moreover, compared to participants who consumed salt-preserved vegetables almost daily, those who rarely or never consumed had a higher eGFR.

Our findings agree with those of previous studies that examined the effect of plant foods on kidney function. A cohort study of 432,732 individuals from a UK biobank demonstrated that higher vegetable intake was associated with a higher eGFR, with a causal link between them [21]. In a Japanese study of 2,755 participants, moderate vegetable juice consumption was associated with a lower decline in eGFR compared to the rare consumption group [22]. Another study in Japanese adults depicted that dietary patterns characterized by vegetables, fish, fruit, bean products, and rice were inversely associated with annual changes in eGFR [23]. These studies demonstrate that a plant-based diet has a renal-protective effect. Our findings extended the evidence of the beneficial effects of vegetable intake on kidney function in the elderly population.

Several mechanisms may explain the relationship between vegetables and renal function. Oxidative stress is the primary mechanism involved in kidney disease progression. Vegetables are rich in fiber, bioactive phytochemicals, and antioxidants that may reduce the levels of classic inflammatory biomarkers such as C-reactive protein (CRP) [24]. According to a multi-ethnic atherosclerosis cohort study, a diet rich in fruits, vegetables, whole grains, and fish had an inverse association with inflammatory indicators, including CRP and E-selectin [24, 25]. Our study additionally indicates that individuals who maintain a regular vegetable consumption pattern exhibit lower levels of hs-CRP compared to those with sporadic vegetable consumption habits. It is of particular interest that hs-CRP exhibits a negative correlation with estimated eGFR, further highlighting the potential implications of dietary choices on renal health. Additionally, an acid-producing diet has been reported to exert a severe halt on kidney function [9, 26], while low-to-moderate certainty evidence suggests that alkali supplementation slows down the rate of kidney function decline in CKD patients [27]. Vegetables are abundant in alkaline ions and are therefore considered to prevent chronic acidosis, which is beneficial for kidney function. In a randomized controlled study of stage 3 CKD patients, compared with patients with usual care, those with vegetable and fruit or bicarbonate supplementation had a lesser eGFR decline [28]. Consequently, dietary supplementation with alkaline food may preserve eGFR. Furthermore, individuals who incorporate a higher frequency of vegetables into their dietary intake may exhibit a greater propensity for maintaining healthier lifestyles and a heightened awareness and willingness to proactively manage chronic diseases such as hypertension and diabetes, both of which have significant implications for kidney function. Within the scope of this study, our findings also revealed an association between regular vegetable consumption and lowered diastolic blood pressure as well as reduced blood glucose levels.

This study also observed that rarely/never consumption of salt-preserved vegetables was associated with a higher eGFR. Our results agree with the conclusions of the Chang Gung Memorial Hospital study, which indicated that excessive salt-preserved food was positively associated with rapid renal function decline in diabetic patients [16]. Generally, salt-preserved vegetables contain high levels of salt. Higher dietary sodium intake has a devastating effect on kidney function and is associated with CKD development [29]. The potential mechanisms might include increased oxidative stress by decreasing the renal expression of superoxide dismutase and modulating renal transforming growth factor-band nitric oxide by directly affecting the endothelium [30, 31]. However, in the Korean genome and epidemiology study, fermented vegetable intake revealed insignificant association with incidents of a decline in eGFR (eGFR < 60 mL/min/1.73 m^2^)^[5]^. In addition to high sodium intake, the author speculated that the fermented vegetables contained healthy microbes, such as *lactobacilli* and *bifidobacterium*. CKD patients tend to have a decreased percentage of beneficial microbes in the gut; hence, pre- or probiotic supplementation improves kidney functions [5, 32]. Therefore, probiotics exert a protective effect that may counteract the damage caused by high sodium intake. However, no significant association between occasional consumption of salt-preserved vegetables and eGFR was observed in this study.

In addition, the results were recombined from the data of two variables: fresh and salt-preserved vegetables. The results indicated that compared to people with two risk factors (low fresh vegetable intake and high salt-vegetables intake), those with only one risk factor or no risk factors demonstrated a higher eGFR. In addition, the association between eGFR with fresh vegetables were significant among people consuming salt-preserved vegetables almost daily. In this study, 13.4% of the elderly reported a daily intake of salt-preserved vegetables. Salt-preserved vegetables are widely regarded in Chinese culture as components of a lifestyle and are often consumed by the elderly [33].Future studies using more refined measures that consider both the quantity and frequency of salt-preserved vegetable intake are warranted to examine their effects on kidney function.

This study also encountered certain limitations. First, measures on fresh and salt-preserved vegetable intakes were coarse which relied on a few questions regarding consumption frequency. Detailed information regarding these quantities should be collected. Admittedly, this crude categorization is subjective, and self-reported dietary intake may be subject to recall bias and reporting errors. Additionally, this study had a relatively small sample size and short follow-up period, which may restrict the decline in eGFR. This could have underestimated the association between fresh vegetable intake, salt-preserved vegetable intake, and eGFR. Chronic diseases were self-reported; hence, their prevalence was relatively low. Therefore, residual confounding may persist in these associations after adjusting for chronic diseases. Finally, because vegetable intake, especially salt-preserved vegetables, represents only a few parts of China, the findings might not be generalizable to other populations.

In conclusion, this study observed an additive effect of fresh vegetable intake and salt-preserved vegetable intake on eGFR in the elderly population in China. Our results suggest that limiting salt-preserved vegetable intake and their replacement with fresh or other forms of preserved vegetables should be encouraged while promoting healthy vegetable consumption. This conclusion must be validated by future studies, such as those investigating the effects of the quantity of fresh vegetables and salt-preserved vegetables on the degree of kidney function in longitudinal follow-ups.

## Data Availability

All the datasets analyzed in this study are available in the website of the CLHLS (https://opendata.pku.edu.cn/dataverse/CHADS).

## References

[1] Jankowski J, Floege J, Fliser D, Bohm M, Marx N (2021). Cardiovascular Disease in chronic kidney disease: pathophysiological insights and therapeutic options. Circulation.

[2] Coresh J, Turin TC, Matsushita K, Sang Y, Ballew SH, Appel LJ, Arima H, Chadban SJ, Cirillo M, Djurdjev O (2014). Decline in estimated glomerular filtration rate and subsequent risk of end-stage renal disease and mortality. JAMA.

[3] Peralta CA, Shlipak MG, Judd S, Cushman M, McClellan W, Zakai NA, Safford MM, Zhang X, Muntner P, Warnock D (2011). Detection of chronic kidney disease with creatinine, cystatin C, and urine albumin-to-creatinine ratio and association with progression to end-stage renal disease and mortality. JAMA.

[4] Kalantar-Zadeh K, Fouque D (2017). Nutritional Management of chronic kidney disease. N Engl J Med.

[5] Jhee JH, Kee YK, Park JT, Chang TI, Kang EW, Yoo TH, Kang SW, Han SH (2019). A Diet Rich in vegetables and Fruit and Incident CKD: A Community-Based prospective cohort study. Am J Kidney Dis.

[6] Asghari G, Momenan M, Yuzbashian E, Mirmiran P, Azizi F (2018). Dietary pattern and incidence of chronic kidney disease among adults: a population-based study. Nutr Metab (Lond).

[7] Rysz J, Franczyk B, Cialkowska-Rysz A, Gluba-Brzozka A. The Effect of Diet on the survival of patients with chronic kidney disease. Nutrients 2017, 9(5). 10.3390/nu9050495 PMC5452225 28505087

[8] Goraya N, Simoni J, Jo CH, Wesson DE (2013). A comparison of treating metabolic acidosis in CKD stage 4 hypertensive kidney disease with fruits and vegetables or sodium bicarbonate. Clin J Am Soc Nephrol.

[9] Goraya N, Simoni J, Jo C, Wesson DE (2012). Dietary acid reduction with fruits and vegetables or bicarbonate attenuates kidney injury in patients with a moderately reduced glomerular filtration rate due to hypertensive nephropathy. Kidney Int.

[10] Mahajan A, Simoni J, Sheather SJ, Broglio KR, Rajab MH, Wesson DE (2010). Daily oral sodium bicarbonate preserves glomerular filtration rate by slowing its decline in early hypertensive nephropathy. Kidney Int.

[11] Herber-Gast GM, Boersma M, Verschuren WMM, Stehouwer CDA, Gansevoort RT, Bakker SJL, Spijkerman AMW (2017). Consumption of whole grains, fruit and vegetables is not associated with indices of renal function in the population-based longitudinal Doetinchem study. Br J Nutr.

[12] He FJ, Jenner KH, Macgregor GA (2010). WASH-world action on salt and health. Kidney Int.

[13] Sugiura T, Takase H, Ohte N, Dohi Y (2018). Dietary salt intake is a significant determinant of impaired kidney function in the General Population. Kidney Blood Press Res.

[14] Malta D, Petersen KS, Johnson C, Trieu K, Rae S, Jefferson K, Santos JA, Wong MMY, Raj TS, Webster J (2018). High sodium intake increases blood pressure and risk of kidney disease. From the Science of Salt: a regularly updated systematic review of salt and health outcomes (August 2016 to March 2017). J Clin Hypertens (Greenwich).

[15] Ohta Y, Tsuchihashi T, Kiyohara K, Oniki H (2013). High salt intake promotes a decline in renal function in hypertensive patients: a 10-year observational study. Hypertens Res.

[16] Lin CW, Chen IW, Lin YT, Chen HY, Hung SY. Association of unhealthy dietary behaviors with renal function decline in patients with diabetes. BMJ Open Diabetes Res Care 2020, 8(1). 10.1136/bmjdrc-2019-000743 PMC6954781 31958295

[17] Sharma S, McFann K, Chonchol M, de Boer IH, Kendrick J (2013). Association between dietary sodium and potassium intake with chronic kidney disease in US adults: a cross-sectional study. Am J Nephrol.

[18] Zeng Y, Feng Q, Hesketh T, Christensen K, Vaupel JW (2017). Survival, disabilities in activities of daily living, and physical and cognitive functioning among the oldest-old in China: a cohort study. Lancet.

[19] Lv Y, Mao C, Yin Z, Li F, Wu X, Shi X (2019). Healthy ageing and biomarkers Cohort Study (HABCS): a cohort profile. BMJ Open.

[20] Levey AS, Stevens LA, Schmid CH, Zhang YL, Castro AF, Feldman HI, Kusek JW, Eggers P, Van Lente F, Greene T (2009). A new equation to estimate glomerular filtration rate. Ann Intern Med.

[21] Park S, Lee S, Kim Y, Lee Y, Kang MW, Kim K, Kim YC, Han SS, Lee H, Lee JP (2021). Observational or genetically predicted higher vegetable intake and kidney function impairment: an Integrated Population-Scale cross-sectional analysis and mendelian randomization study. J Nutr.

[22] Fujii R, Kondo T, Tsukamoto M, Kawai S, Sasakabe T, Naito M, Kubo Y, Okada R, Tamura T, Hishida A (2021). Association of self-reported moderate vegetable juice intake with small decline in kidney function in a five-year prospective study. Nutrition.

[23] Ma E, Ohira T, Yasumura S, Nakano H, Eguchi E, Miyazaki M, Hosoya M, Sakai A, Takahashi A, Ohira H et al. Dietary patterns and progression of impaired kidney function in japanese adults: a longitudinal analysis for the Fukushima Health Management Survey, 2011–2015. Nutrients 2021, 13(1). 10.3390/nu13010168 PMC7827845 33430501

[24] Whalen KA, McCullough ML, Flanders WD, Hartman TJ, Judd S, Bostick RM (2016). Paleolithic and Mediterranean Diet Pattern Scores are inversely Associated with biomarkers of inflammation and oxidative balance in adults. J Nutr.

[25] Nettleton JA, Steffen LM, Mayer-Davis EJ, Jenny NS, Jiang R, Herrington DM, Jacobs DR (2006). Dietary patterns are associated with biochemical markers of inflammation and endothelial activation in the multi-ethnic study of atherosclerosis (MESA). Am J Clin Nutr.

[26] Rebholz CM, Coresh J, Grams ME, Steffen LM, Anderson CA, Appel LJ, Crews DC (2015). Dietary acid load and incident chronic kidney disease: results from the ARIC Study. Am J Nephrol.

[27] Navaneethan SD, Shao J, Buysse J, Bushinsky DA (2019). Effects of treatment of metabolic acidosis in CKD: a systematic review and Meta-analysis. Clin J Am Soc Nephrol.

[28] Goraya N, Simoni J, Jo CH, Wesson DE (2014). Treatment of metabolic acidosis in patients with stage 3 chronic kidney disease with fruits and vegetables or oral bicarbonate reduces urine angiotensinogen and preserves glomerular filtration rate. Kidney Int.

[29] Yoon CY, Noh J, Lee J, Kee YK, Seo C, Lee M, Cha MU, Kim H, Park S, Yun HR (2018). High and low sodium intakes are associated with incident chronic kidney disease in patients with normal renal function and hypertension. Kidney Int.

[30] Meng S, Roberts LJ, Cason GW, Curry TS, Manning RD (2002). Superoxide dismutase and oxidative stress in Dahl salt-sensitive and -resistant rats. Am J Physiol Regul Integr Comp Physiol.

[31] Ying WZ, Sanders PW (1999). Dietary salt increases endothelial nitric oxide synthase and TGF-beta1 in rat aortic endothelium. Am J Physiol.

[32] Xu KY, Xia GH, Lu JQ, Chen MX, Zhen X, Wang S, You C, Nie J, Zhou HW, Yin J (2017). Impaired renal function and dysbiosis of gut microbiota contribute to increased trimethylamine-N-oxide in chronic kidney disease patients. Sci Rep.

[33] A Survey Report on Centenarians. in Jiangsu Province. [(accessed on 17 July 2015)]. Available online: https://wenku.baidu.com/view/2ea870f45322aaea998fcc22bcd126fff7055d11.html.

